# Editorial: The evolution, diversity, and functions of alternative splicing (AS) in plant responses to abiotic stress

**DOI:** 10.3389/fpls.2023.1170258

**Published:** 2023-03-22

**Authors:** Fang Bai, Enamul Huq, Justyna Jadwiga Olas

**Affiliations:** ^1^ University of Florida, Gainesville, FL, United States; ^2^ The University of Texas at Austin, Austin, TX, United States; ^3^ University of Potsdam, Potsdam, Germany

**Keywords:** alternative splicing, AS, abiotic stress, plant response, evolution, diversity and function

Alternative splicing (AS) is a widespread mechanism in eukaryotic gene regulation that enables the generation of multiple protein isoforms from a single gene. AS can result in the inclusion or exclusion of specific exons, alternative 5’ or 3’ splice site selection, or intron retention. The diversity of mRNA isoforms produced through AS greatly expands the proteome and allows for finer regulation of gene expression. In plants, AS plays a critical role in the response to abiotic stress, including drought, high salinity, extreme temperatures, and nutrient deficiency. Abiotic stress can significantly alter gene expression, and AS provides a mechanism for rapid and precise regulation of gene expression under changing environmental conditions. In addition to regulating gene expression, AS also contributes to the evolution of new genes and functions. By generating novel protein isoforms, AS can produce new functions that can help plants adapt to changing environments. Global climate change is bringing new challenges to natural ecosystems around the world. Increasing temperatures and soil salinity levels are contributing to plant abiotic stress, with direct consequences for agricultural crop production. In plants, alternative splicing is of growing importance as more genes are found to undergo AS, which may impact strategies for improving plant phenotypes. In this Research Topic, we aimed to increase the understanding of how alternative splicing modulates plant development and the response to environmental stress. In the following, key ideas contributed to this Research Topic are summarized which serve to illustrate the broad and complex ways that improve the adaption of plant growth under abiotic stresses.

In a review paper, Rosenkranz et al. provide an overview of the impact of heat stress on plants and their response to it, with a focus on the role of AS in regulating plant survival and acclimation to stress conditions. AS events, such as intron retention, can impact the transcriptome and protein synthesis and have been linked to thermotolerance in some cases. The authors highlight the complex nature of splicing, which is affected by multiple factors including RNA polymerase II elongation rate, chromatin structure, and histone and DNA modifications. The authors discussed the role of proteins involved in splicing regulation, including snRNPs, SR proteins, and hnRNPs. The authors reviewed the recent progress on studying the interplay between epigenetic modifications, RNA structure, and splicing regulation. Changes in histone modifications and DNA methylation can impact gene expression regulation and splicing profiles. The authors suggest that similar mechanisms, such as RNA thermometers, could be present in plants, although further research is required. The authors emphasize the significance of AS in regulating plant stress response and thermotolerance, but also raise questions about the balance between the benefits and drawbacks of AS in this context.

Yang et al. focus on investigating the alternative splicing (AS) landscape of *Brassica napus* in response to different abiotic stresses, including dehydration, cold, NaCl, and ABA. The authors analyzed RNA-seq data from 26 samples of the cultivar *Zhongshuang 11* to quantify the transcript per million (TPM) of each isoform and to identify the frequency of different types of AS events. The results showed that intron retention was the most common AS event, while exon skipping was the least frequent. The authors also identified 357 differentially AS genes (DAS genes) across different treatments, of which 81 were induced by multiple stresses. The study found that the majority of the DAS genes were involved in splicing and metabolic processes. This study provides new insights into the AS landscape of *Brassica napus* in response to different abiotic stresses and highlights the potential role of DAS genes in stress responses.

Wu et al. studied the genomic structure, gene sequence, and protein isoforms of three splicing variants of *TaNAK1*, *TaNAK1.1*, *TaNAK1.2*, and *TaNAK1.3* in wheat. *TaNAK1* is a target gene of a wheat specific-miRNA *tae-miR5048*. The authors compared the gene sequence with the three splicing variants and discovered that each variant has a different number of deleted base pairs in the coding sequence, which results in truncated protein isoforms with different amino acid lengths. The authors also explored the expression patterns of the *TaNAK1* variants in different developmental stages and tissues/organs of wheat and found that only two variants, *TaNAK1.1* and *TaNAK1.2*, were detected and exhibit different expression patterns across ten tissues/organs. This study provides new insights into the genomic structure, gene sequence, and protein isoforms of *TaNAK1* and its splicing variants in wheat. The results suggest that the two variants *TaNAK1.1* and *TaNAK1.2* might play important roles in wheat growth and development and warrant further investigation.

The study by Martin delves into the intricate regulation of gene expression in response to retrograde signals from chloroplasts and light signals, with a focus on post-transcriptional regulation through alternative splicing. The results show that alternative splicing mimics transcriptional responses triggered by retrograde signals and involves *GUN1* and the non-sense mediated decay pathway in downregulating the expression of chloroplast proteins. The study highlights the importance of understanding the interplay between light signals and retrograde signaling-regulated splicing. Using vast-tools, the authors identified 252 differentially spliced events in response to norflurazon. The inclusion of an alternative sequence was greater in 165 out of 252 events in the presence of norflurazon, and the splicing changes were milder in the *gun1* mutant. The study also compared the splicing events between seedlings treated or not with lincomycin and found similar regulation, suggesting that retrograde signals control splicing of a subset of transcripts in a *GUN1*-dependent manner. This study identifies alternative splicing as a powerful mechanism that mimics transcriptional responses and the role of *GUN1* in triggering downstream alternative splicing regulation.

The collection of articles in this Research Topic demonstrates that AS is a critical mechanism in the response of plants to abiotic stress, providing a means of rapid and precise regulation of gene expression. We hope that the knowledge can facilitate further success of new studies and breeding for abiotic stress-resistance plants. Future studies will undoubtedly continue to unravel the complexity of AS in plants and its critical role in plant adaptation to environmental stress.

## Author contributions

FB initiated this Research Topic. For the Editorial, all authors reviewed all Research Topic articles. FB summarized the reviews and drafted the first manuscript. EH revised and modified it to get the final version. All authors approved it for publication. 

